# Disentangling the impact of motion artifact correction algorithms on functional near-infrared spectroscopy–based brain network analysis

**DOI:** 10.1117/1.NPh.11.4.045006

**Published:** 2024-10-23

**Authors:** Shuo Guan, Yuhang Li, Yuxi Luo, Haijing Niu, Yuanyuan Gao, Dalin Yang, Rihui Li

**Affiliations:** a University of Macau, Institute of Collaborative Innovation, Center for Cognitive and Brain Sciences, Taipa, Macau S.A.R., China; b University of Macau, Department of Psychology, Faculty of Social Science, Taipa, China; c Sun Yat-Sen University, School of Biomedical Engineering, Shenzhen, China; d Beijing Normal University, IDG/McGovern Institute for Brain Research, State Key Laboratory of Cognitive Neuroscience and Learning, Beijing, China; e Stanford University, Center for Interdisciplinary Brain Sciences Research, Department of Psychiatry and Behavioral Sciences, Stanford, California, United States; f Washington University School of Medicine, Mallinckrodt Institute of Radiology, St. Louis, Missouri, United States; g University of Macau, Department of Electrical and Computer Engineering, Faculty of Science and Technology, Taipa, China

**Keywords:** functional connectivity, functional near-infrared spectroscopy, motion artifact, brain network

## Abstract

**Significance:**

Functional near-infrared spectroscopy (fNIRS) has been widely used to assess brain functional networks due to its superior ecological validity. Generally, fNIRS signals are sensitive to motion artifacts (MA), which can be removed by various MA correction algorithms. Yet, fNIRS signals may also undergo varying degrees of distortion due to MA correction, leading to notable alternation in functional connectivity (FC) analysis results.

**Aim:**

We aimed to investigate the effect of different MA correction algorithms on the performance of brain FC and topology analyses.

**Approach:**

We evaluated various MA correction algorithms on simulated and experimental datasets, including principal component analysis, spline interpolation, correlation-based signal improvement, Kalman filtering, wavelet filtering, and temporal derivative distribution repair (TDDR). The mean FC of each pre-defined network, receiver operating characteristic (ROC), and graph theory metrics were investigated to assess the performance of different algorithms.

**Results:**

Although most algorithms did not differ significantly from each other, the TDDR and wavelet filtering turned out to be the most effective methods for FC and topological analysis, as evidenced by their superior denoising ability, the best ROC, and an enhanced ability to recover the original FC pattern.

**Conclusions:**

The findings of our study elucidate the varying impact of MA correction algorithms on brain FC analysis, which could serve as a reference for choosing the most appropriate method for future FC research. As guidance, we recommend using TDDR or wavelet filtering to minimize the impact of MA correction in brain network analysis.

## 1 Introduction

Functional connectivity (FC) characterizes the temporal synchronization of neuronal activities among different anatomically separated brain regions.^1^^,^^2^ By employing functional imaging techniques such as functional magnetic resonance imaging (fMRI) and functional near-infrared spectroscopy (fNIRS), FC analysis can advance our understanding of aberrant neural circuits associated with brain disorders.^3^^,^^4^ Specifically, aberrant FC patterns have emerged as key indicators in diseases such as Alzheimer’s disease, autism spectrum disorders, and schizophrenia, revealing their significance not just in identifying these conditions but also in tailoring treatment approaches and tracking disease progression.^5^^–^^7^

Despite being less commonly associated with high-definition exploration of brain network compared with fMRI, portable fNIRS possesses unique strengths in mapping naturalistic functions of the brain,^8^^,^^9^ making it a particularly suitable technique for investigating brain network dynamics under more ecological settings. In a routine acquisition of fNIRS data, however, motion artifact (MA) is a critical challenge that severely affects the signal’s fidelity. These artifacts, defined as unexpected changes in recorded signals due to subject movement, can drastically reduce the signal-to-noise ratio, thus interfering with the accurate interpretation of neural activities.^10^ The consequences are twofold: MAs can either introduce spurious components that mimic neural activity (false positives) or obscure actual neural activations (false negatives), both of which are detrimental to the reliability of neuroscience findings.

Over the last two decades, there have been numerous proposed methods for addressing MAs in fNIRS measurements. Classical approaches such as spline interpolation, wavelet filtering, and principal component analysis (PCA) have been widely used in fNIRS studies.^11^ New progress in MA removal was also made recently by the so-called temporal derivative distribution repair (TDDR), an effective approach that takes into account the temporal characteristic of MA.^12^ Deep learning techniques were also recently introduced to remove MAs in fNIRS data.^13^ Although these studies have explored the efficacy of multiple MA methods in improving task-related brain activation mapping,^14^ the impact of these MA removal methods on brain FC analysis remains largely unknown. Assessing the impact of various MA correction methods on FC analysis is therefore critical for establishing a reference for enhancing the reliability of brain FC analysis.

To bridge this gap, the present study systematically evaluated how various MA correction methods affect brain FC analysis by analyzing simulated and experimental datasets. Specifically, we thoroughly compared the performance of six commonly used MA correction techniques, namely, PCA, spline interpolation, wavelet filtering, Kalman filtering, correlation-based signal improvement (CBSI), and TDDR.^11^^,^^15^^–^^17^ Sensitivity–specificity analysis in conjunction with receiver operating characteristic (ROC) curves was employed to test the efficacy and accuracy of each method. The primary objective of this study was to gain a comprehensive understanding of the impact of MA removal techniques on typical FC analysis. We aimed to provide a robust reference for selecting preprocessing methods in future fNIRS studies, thereby facilitating a more precise interpretation of the brain connectome pattern under investigation.

## 2 Methods and Materials

### 2.1 Motion Artifact Correction Algorithms

#### 2.1.1 Principal component analysis

PCA is utilized in targeted PCA to remove systemic interference from fNIRS signals.^18^ This approach assumes that physiological noises, such as those from cardiovascular and respiratory sources, dominate the signal variance during baseline conditions and hence can be isolated as the principal components (PCs) with the highest variance. The order of these components is related to the proportion of variance in the original data that each component accounts for. Thus, the first component will account for the largest proportion of the data variance. As the amplitudes of motion artifacts are typically much larger than normal physiological fNIRS signals, they should constitute a large portion of the data variance. The first few PCs that are considered to represent systematic interference are then discarded to retain the brain signal information. This is achieved by reconstructing the signal from the remaining components after removing those that account for the majority of the variance (and are considered to be noise). The procedure involves the Eigen-decomposition of the spatial correlation matrix from baseline signals. By projecting the signal onto the orthogonal complement of the PCs representing the noise, cleaner fNIRS signals theoretically indicative of neural activity are obtained.

#### 2.1.2 Spline interpolation

The motion artifact reduction algorithm (MARA) using spline interpolation is a two-step process devised to mitigate MAs in fNIRS data.^19^ MARA assumes that fNIRS signals are linearly composed of true neurophysiological signals and MAs, the latter becoming dominant during corruption. Firstly, MARA employs the moving standard deviation (MSD) within a time window to detect MAs. The detection hinges on comparing MSD values against a pre-set threshold, identifying the beginnings and ends of artifacts. Non-artifact and artifact segments are then defined. Second, spline interpolation fits the artifact segments. Subtracting this fit from the actual data yields a potential true signal without MAs. This difference, however, might differ in magnitude from the non-corrupted segments. Finally, level correction of the entire signal is required to account for the differences in the mean values among consecutive segments. Spline interpolation requires precise artifact detection and level correction, complicating real-time filtering. Yet, it is widely used for offline analysis and supported by open-source fNIRS toolboxes. Variants of spline interpolation have been proposed to refine the method’s efficacy.^20^

#### 2.1.3 Wavelet filtering

The wavelet-based method is a technique for removing MAs from fNIRS signals without additional equipment.^21^ It operates under the principle that the measured signal is a composition of the underlying physiological signal and noise. Utilizing discrete wavelet transform, signals are decomposed into coefficients reflecting different frequencies. These coefficients are calculated using scaling and wavelet functions

$$y(t)=\underset{k}{\sum }c_{j_{0}k}\phi_{j_{0}k}(t)+\underset{k}{\sum }\overset{\infty }{\underset{j=j_{0}}{\sum }}d_{jk}\psi_{jk}(t).$$ (1)

Here, $\phi_{jk}(t)$ and $\psi_{jk}(t)$ are the scaling and functions and $c_{jk}$ and $d_{jk}$ are the approximation and detail coefficients, respectively. j and k are the wavelet dilation and translation parameters, respectively, with $j_{0}$ the coarsest decomposition.

Artifacts are identified by analyzing the probability distribution of each wavelet coefficient against a threshold. Coefficients considered as dominated by artifacts are set to zero. After thresholding, an inverse transform reconstructs the filtered signal. The method’s efficiency relies on appropriate threshold tuning and can process optical intensities, densities, and concentration changes.

#### 2.1.4 Kalman filtering

The Kalman filter has been proposed for MA removal in fNIRS data structured around an autoregressive (AR) model to represent the artifact-free fNIRS signal. The coefficients of the AR model are determined using historical data and the Yule–Walker equations. The Kalman filter operates using a state space model, with the process equation predicting the current state based on previous ones plus noise, whereas the measurement equation relates the current state to the observed signal with additional noise.^22^

$$\phi (n)=A\phi (n-1)+\omega_{n},\;\phi (n)=[x(n)]\cdots x(n-p+1){]}^{T},$$ (2)

where ϕ(n) consists of p artifact-free fNIRS signals, and $\omega_{n}$ represents the zero-mean noise in the AR model. The output measurements of the Kalman filter are assumed to be motion-corrupted signals, whereas the states x are motion-free signals and the measurement noise is motion artifacts. The covariance of the measurement noise is calculated as the variance of the entire data series, whereas the covariance of the process noise is calculated as the variance of the motion-free segments.

#### 2.1.5 Correlation-based signal improvement

The CBSI method was introduced to address MAs in fNIRS data by exploiting the typically negative correlation between the oxygenated hemoglobin (HbO) and deoxygenated hemoglobin (HbR) concentrations.^23^ CBSI assumes a strictly negative correlation between HbO and HbR, consistent MA impact on both, and no correlation between MAs and actual concentration changes. CBSI mathematically models the relationship between HbO and HbR and eliminates MAs, resulting in cleaned signals for HbO and HbR.

$$\left\{ \begin{array}{l} x=x_{0}+\alpha *F+\text{Noise}\\ y=y_{0}+F+\text{Noise} \end{array}.\right.$$ (3)

The observed HbO and HbR signal, denoted as x and y, are assumed to be composed of contributions from the estimated signal $x_{0}$ and $y_{0}$, the motion artifact F that affects both HbO and HbR similarly but with different scaling factors captured by the constant α=std(x)/std(y), and instrument noise.

#### 2.1.6 Temporal derivative distribution repair

The TDDR is an online method for filtering fNIRS signals; assuming non-motion fluctuations are normally distributed, most fluctuations are independent of MAs, and the derivatives of MAs dominate when present.^12^ If these assumptions are met, the contribution from motion will be large and infrequent and therefore lie in the far tail of the normal distribution of signal fluctuations. One possible way to address this problem is to reduce the weight of abnormally large fluctuations. The TDDR algorithm uses a robust estimator to compute observation weights for temporal derivatives of fNIRS signals. It then integrates the weighted and adjusted temporal derivatives to reconstruct the filtered signal. The method works in real time by updating the sum of weighted derivatives in each iteration.

### 2.2 Preparation of fNIRS Data

To assess the effect of different MA correction methods on brain FC analysis, we conducted a sensitivity–specificity analysis on simulated and real fNIRS datasets. Initially, the simulated resting-state fNIRS data with real MA (i.e., shifts and spikes) were employed to validate the capability of various motion correction methods. Subsequently, their effectiveness in motion correction was quantified using actual experimental data, specifically in terms of functional connectivity and topological property. The details of those datasets are explained below.

#### 2.2.1 Simulated fNIRS data

**Fig. 1:**
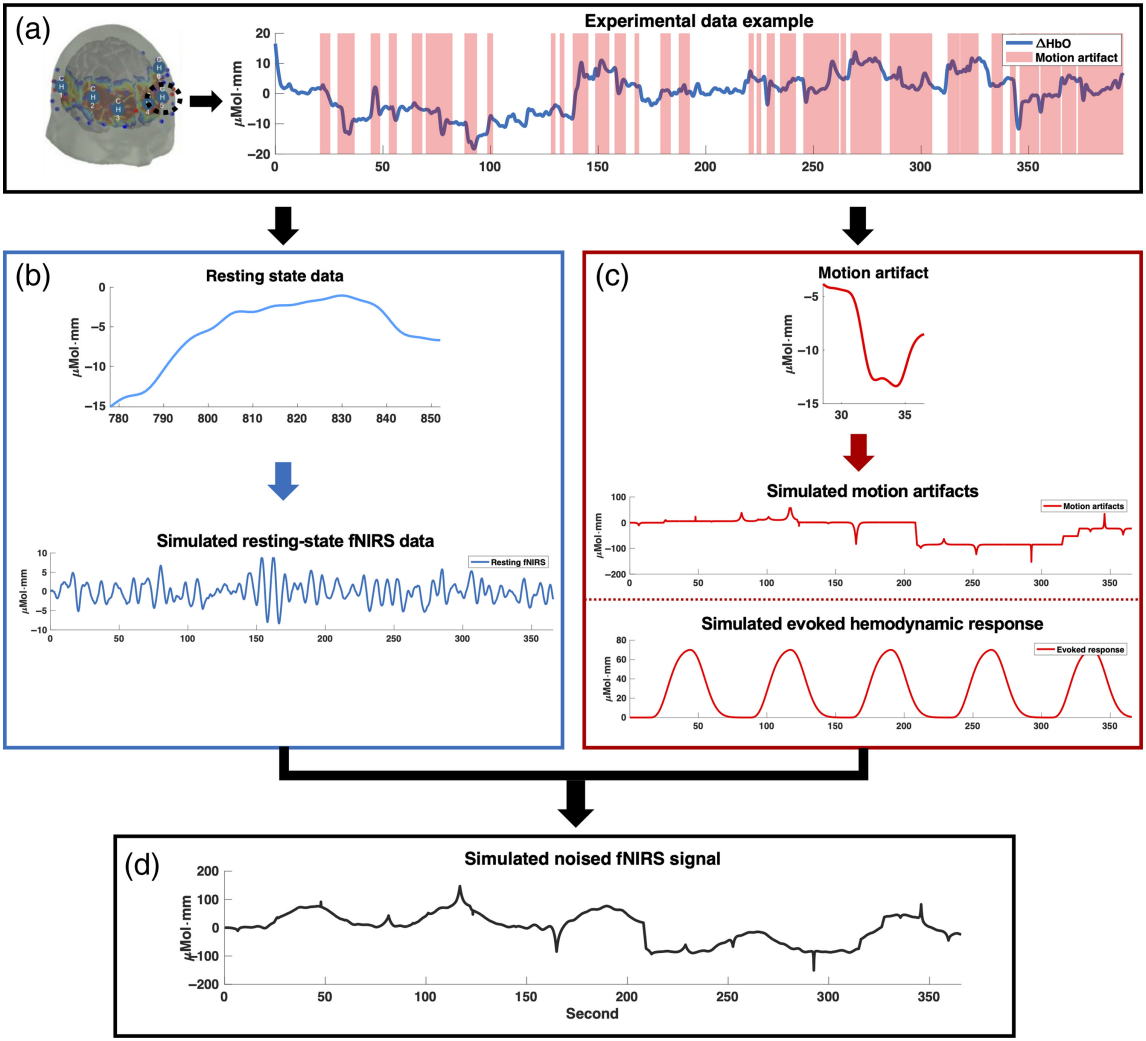
Pipeline of the fNIRS data simulation. (a) fNIRS signal was selected from a specific channel of an experimental dataset. Motion artifact was then identified. (b) Artifact-free, representative resting-state data were segmented and extracted from the selected fNIRS signal, from which artifact-free resting-state signals were simulated. (c) Representative motion artifact was extracted from the selected fNIRS signal, from which a time series with motion artifacts was simulated. In addition, a standard hemodynamic response was also simulated. (d) The noise-affected fNIRS signal was then simulated.

The pipeline of the fNIRS data simulation is summarized in Fig. 1. To simulate six common resting-state brain networks, namely, the default mode network (DMN), frontoparietal network (FPN), sensorimotor network (SMN), visual (VIS), ventral attention network (VAN), and dorsal attention network (DAN), six channels with good signal quality from an open-access multimodal fNIRS resting-state dataset were chosen.^24^ In particular, we deliberately chose channels from diverse brain regions to maximize the likelihood that they would be representative of different brain networks and also to ensure that there was no noise interference among the signals. However, it should be noted that these channels were not precisely and anatomically located within each corresponding network due to the lack of channels covering all six brain networks. Each channel was used to simulate a network consisting of eight channels to achieve high internal correlation, mimicking the tightly coupled activity within each network [i.e., network (NW)]. Data were simulated assuming near-infrared wavelengths of 690 and 830 nm. For each simulation, we generated 300 s of resting-state data at a 7.8-Hz sample rate. The total simulated data (Fig. 1), represented as $F^{\prime }(t)$, are composed of the confluence of three distinct elements: the canonical clean hemodynamic response function (HRF) F(t), the MA component $\Phi_{\mathrm{MA}}(t)$, and the resting-state fNIRS fluctuation term $\Phi_{\mathrm{rs}}(t)$.

$$F^{\prime }(t)=F(t)+\Phi_{\mathrm{MA}}(t)+\Phi_{\mathrm{rs}}(t)+\varepsilon .$$ (4)

We utilized an AR model with five lagged terms to determine the model parameters, which were used to simulate the resting-state fNIRS data $\Phi_{\mathrm{rs}}(t)$.^13^ In Eq. (4), F(t) is designated as the clean HRF. The six amplitudes stipulated for the assessment are furnished as percentages relative to a conventional average amplitude typical of an HRF, namely, 30%, 40%, 50%, 60%, 70%, and 80%.^24^ In the described model, a white noise pattern was assumed with the presence of independent and randomly sampled data points. To simulate MAs commonly observed in fNIRS time series, two distinct types of interference, namely, shift and spike artifacts, were introduced based on a previous study.^25^

The rate of shift artifacts added to the data was around 0.5 per min or 1 shift artifact for every 300 points. The shift artifact was modeled as a scalar shift in amplitude added to the time series sampled from a normal distribution with a zero mean and a standard deviation of five times the original data. Similarly, the rate of spike artifacts added to the data was around 2 per minute. The spike artifact was modeled with a Laplacian distribution function with the peak amplitude sampled from a normal distribution with a mean zero and a standard deviation of five times the standard deviation of the original data. This ensures that the spike artifact occurs at the same time across multiple channels, but the amplitude of the spike artifact varies across the channels. The initial determination of all noise function parameters is based on experimental fNIRS datasets.^24^ Following this, the parameters for each artifact are refined according to the datasets.

#### 2.2.2 Experimental data

**Fig. 2:**
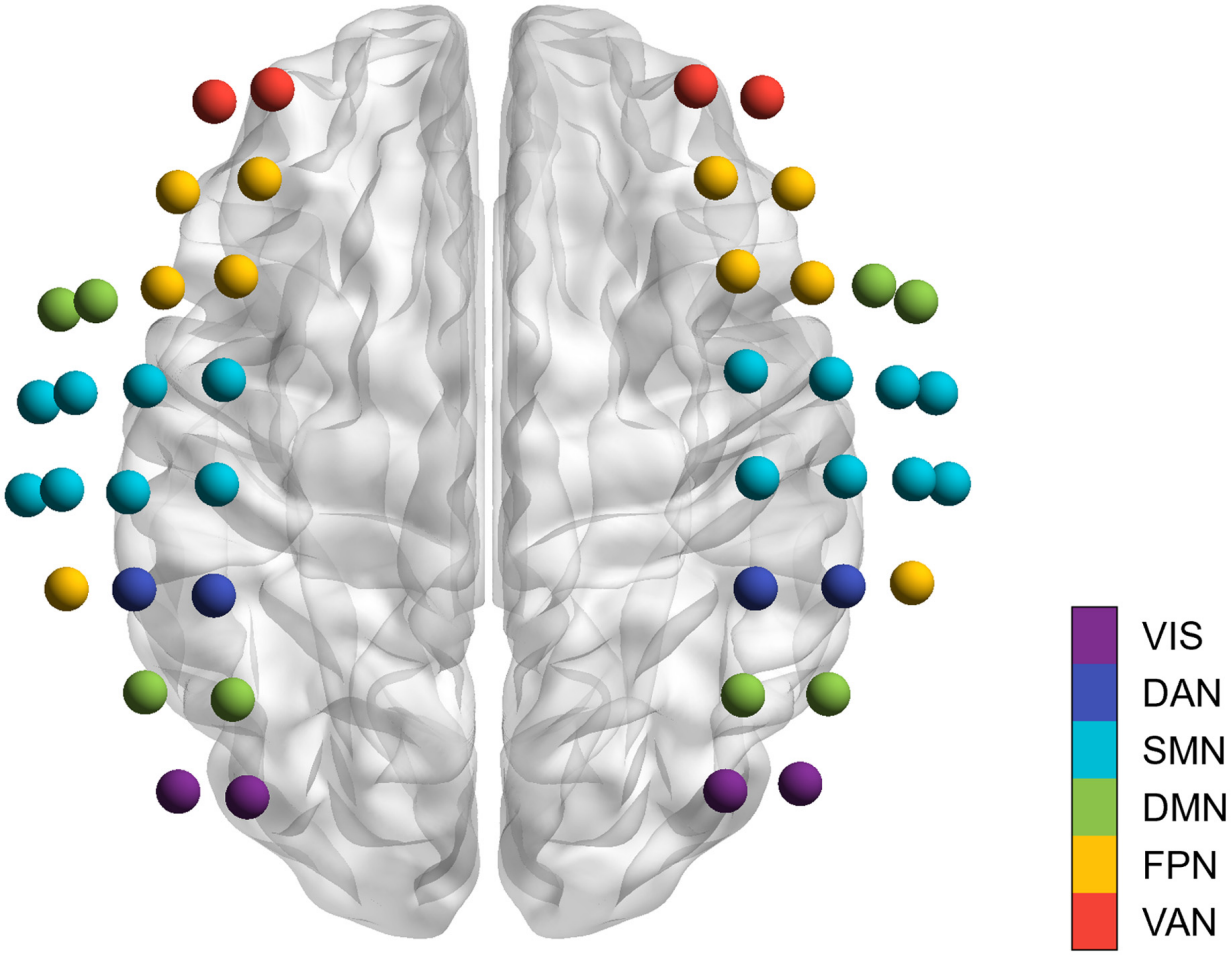
Distribution of the whole-head 46 channels in the experimental dataset. The colored nodes indicated the 46 fNIRS channels, with each color representing different intrinsic brain networks, including the VIS network, DAN, SMN, DMN, FPN, and VAN.

The experimental data utilized in this study were derived from a prior study.^26^ This dataset recorded the HbO from 34 healthy controls (11 males and 20 females, age: 67.61±8.86  years). An ∼11-min resting-state brain scanning session was conducted for each participant with a 46-channel dual-wavelength continuous wave system with a sampling rate of 50 Hz (CW6, TechEn Inc., Milford, Massachusetts, United States). This study was approved by the Medical Research Ethics Committee of XuanWu Hospital. As shown in Fig. 2, the regions of interest covered the six brain networks (i.e., VIS, DAN, SMN, DMN, FPN, and VAN) after overlapping with existing brain parcellation.^27^

### 2.3 Data Preprocessing

The preprocessing and analysis of brain activation were conducted utilizing the NIRS Brain AnalyzIR toolbox.^28^ The raw measured intensity signals were first converted into optical density. Subsequently, the optical density signals were analyzed by different MA correction algorithms, including Kalman filtering, PCA, wavelet filtering, TDDR, and spline interpolation. A bandpass filtering between 0.001 and 0.08 Hz was applied to remove physiological noise. After converting the optical density to the concentration changes of HbO and HbR, the CBSI method was also employed as an independent step of MA correction. Owing to the high sampling rate of the dataset, which leads to a large data volume, we have downsampled data to 7.8 Hz.

### 2.4 Evaluation Criteria and Validation of Functional Connectivity

#### 2.4.1 Functional connectivity

For each participant, we calculated the functional network through Pearson’s correlation analysis between any two fNIRS channels across the entire time series. Due to negative correlations that might arise due to systemic biases or limitations in data processing methods rather than actual changes in brain function,^29^^,^^30^ we only included positive correlations in this study. We further performed a Fisher’s z-transform to calculate the z-value for each correlation coefficient. To statistically compare the variability between different motion correction methods and uncorrected real data, the Friedman test was used, followed by post hoc testing using the Nemenyi test. In addition, false discovery rate (FDR) correction is applied to the post hoc analysis to control the risk of type I errors at a significance level of 0.05.

#### 2.4.2 ROC analysis

Based on the correlation coefficient calculated by Pearson’s correlation as described in the method of Niu’s study,^31^ we further evaluated the performance of each MA removal method through ROC analysis.^32^ We randomly selected two data traces from the same subject’s data or two different data files. We assumed that there is a strong consistency within the subject’s brain signals, which is defined as true positives. There is no significant correlation in the random temporal signals across different subjects, which is defined as false positives.^33^^,^^34^ The ROC analysis was utilized to assess the efficacy of different models in distinguishing between data sourced from the same file and data from different files.^35^ The area under the curve (AUC) metric derived from this ROC analysis provided a quantitative measure of the relative success of various motion correction methods in terms of performance. The ROC analysis was conducted on both the simulated and experimental datasets.

#### 2.4.3 Graph theory–based topological analysis

We also assessed how different MA removal methods affected the graph theory–based topological property of the brain, given that topological analysis has been extensively reported in most FC studies.^4^^,^^36^^,^^37^ For each subject at each sparsity level, we computed the entire network metrics based on an in-house GRETNA package. Specifically, the metrics used in this study included global and local efficiency, small world, and betweenness centrality.^38^ By applying various MA techniques to our dataset, we computed these topological measures and quantitatively assessed the effectiveness of MA removal methods through a series of paired t-tests (corrected by FDR).

## 3 Results

### 3.1 Simulation Results

**Fig. 3:**
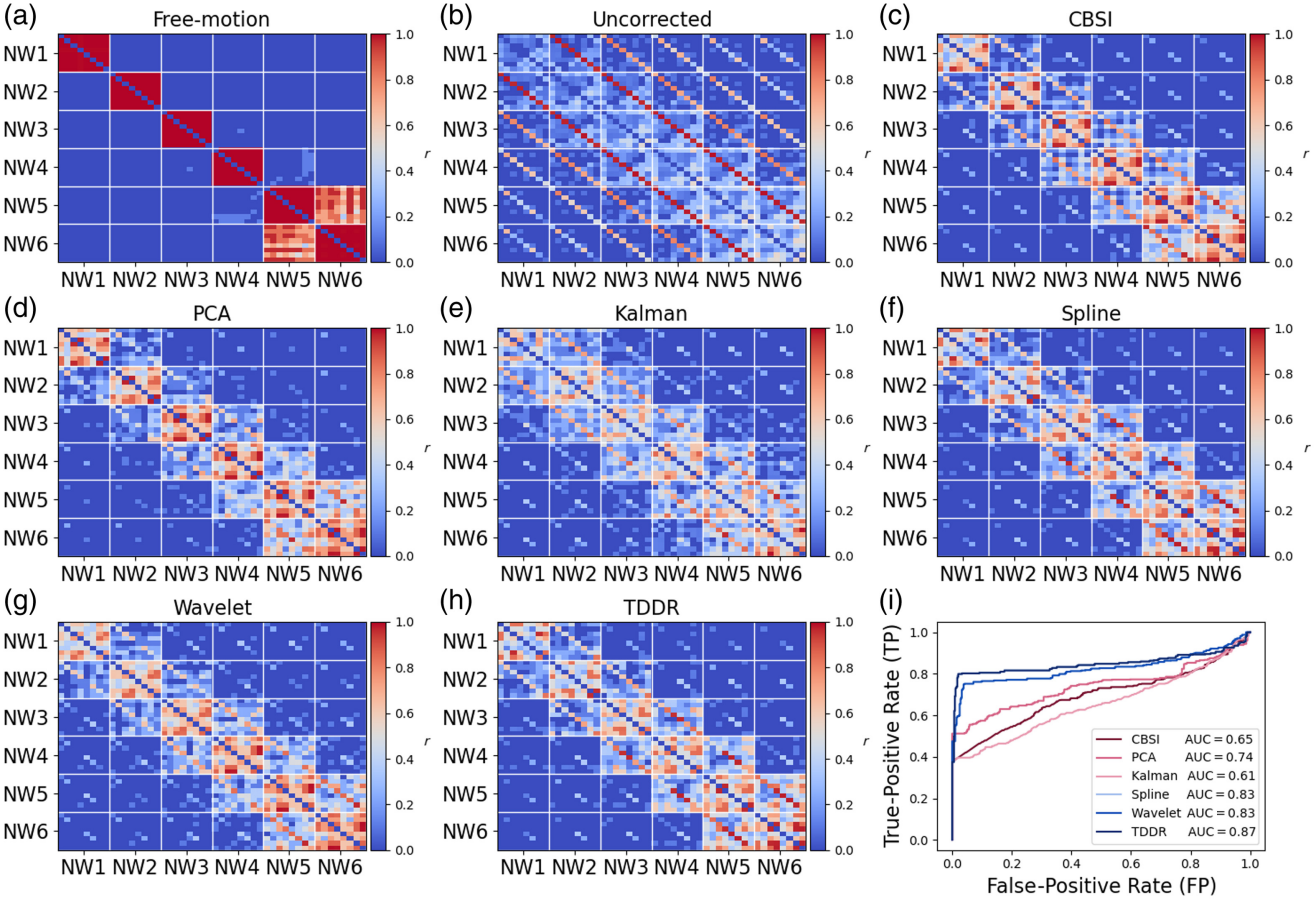
Visualization of the uncorrected and corrected functional connectivity (r) of each NW in the simulated data (a)–(h). (i) Comparison of ROC and AUC obtained from different methods.

The functional connectivity matrices of all six predefined NWs were constructed from the simulated data. As expected, each network showed a strong within-network FC, whereas the FC among these networks was relatively low [Fig. 3(a)]. The within- and between-network FC patterns were altered after MAs were added to the simulated signals [Fig. 3(b)]. Figures 3(c)–3(h) shows the FC matrices after the MAs were corrected by different methods. Quantitively, the wavelet filtering, spline interpolation, PCA, CBSI, and TDDR achieved significant improvements compared with the uncorrected results, whereas Kalman only made a slight improvement [Fig. 3(e)]. The ROC and AUC analysis showed that the TDDR model outperformed all other MA removal methods [AUC = 0.87, Fig. 3(I)], whereas Kalman (AUC = 0.61) demonstrated the lowest AUC among these methods.

**Fig. 4:**
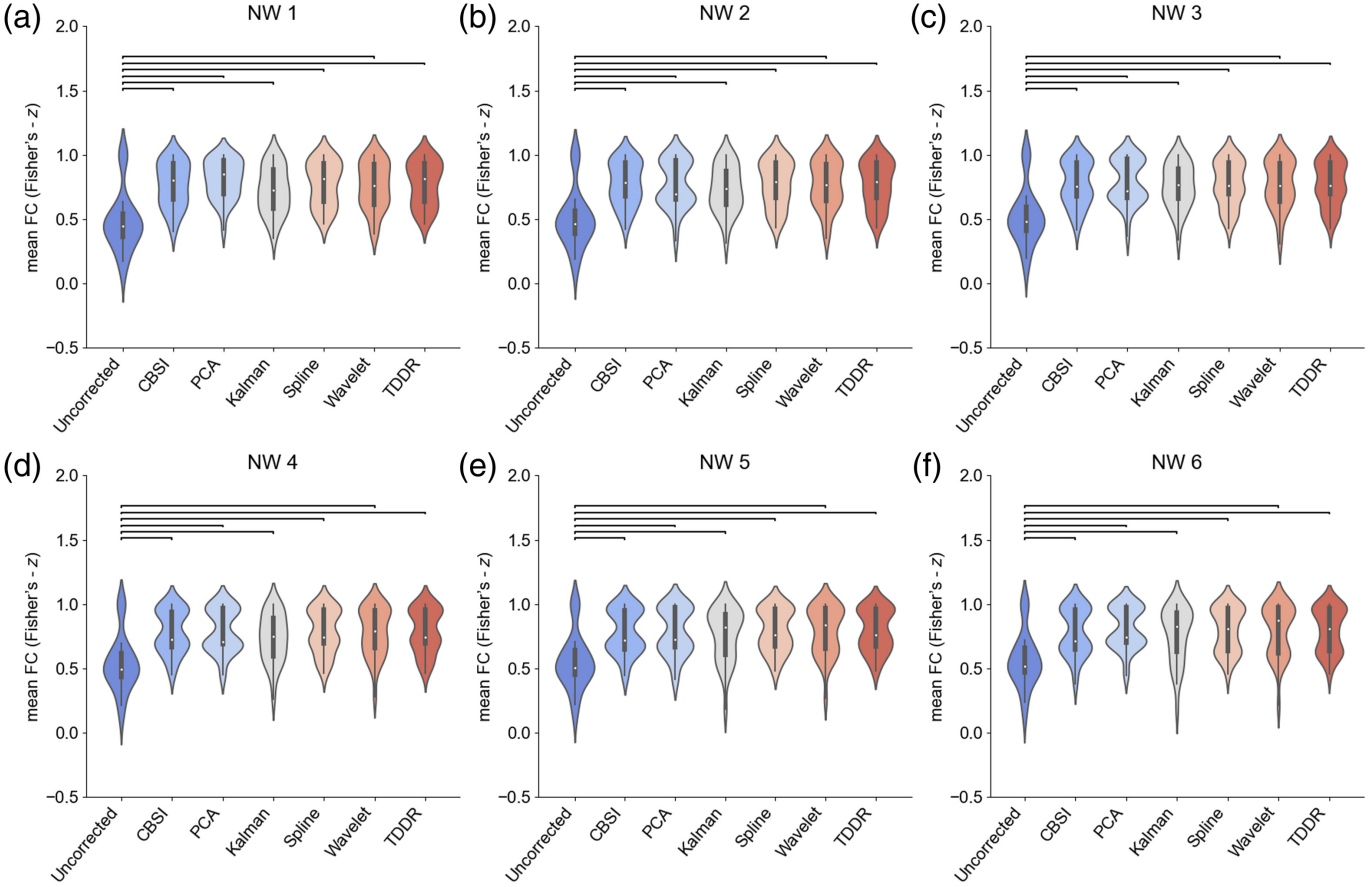
(a)–(f) Statistical comparison of the mean functional connectivity of each NW before and after applying different MA correction methods in the simulated data. Each horizontal line indicates a significant difference between the two methods (p<0.05, FDR-corrected).

As shown in Figs. 4(a)–4(f), significant differences in the mean FC z-value of all simulated networks (p<0.05) were identified. As summarized in Table 1, the mean FC after MA removal by different methods was significantly higher compared with that of the uncorrected data (p<0.01).

**Table 1:** Statistical comparison of the mean within-network FC between the uncorrected simulated data and the corrected simulated data after different correction methods were applied.

Networks	NW1		NW2		NW3		NW4		NW5		NW6	
Uncorrected versus	diff.	p	diff.	p	diff.	p	diff.	p	diff.	p	diff.	p
CBSI	0.29	<0.01	0.27	<0.01	0.26	<0.01	0.25	<0.01	0.23	<0.01	0.22	<0.01
PCA	0.33	<0.01	0.27	<0.01	0.27	<0.01	0.2	<0.01	0.23	<0.01	0.25	<0.01
Kalman	0.24	<0.01	0.23	<0.01	0.24	<0.01	0.25	<0.01	0.21	<0.01	0.2	<0.01
Spline	0.29	<0.01	0.27	<0.01	0.25	<0.01	0.25	<0.01	0.24	<0.01	0.24	<0.01
Wavelet	0.28	<0.01	0.26	<0.01	0.25	<0.01	0.24	<0.01	0.24	<0.01	0.23	<0.01
TDDR	0.29	<0.01	0.27	<0.01	0.25	<0.01	0.25	<0.01	0.24	<0.01	0.24	<0.01

diff., difference of mean FC between the corrected and the uncorrected network; NW, network.

### 3.2 Experimental Results

#### 3.2.1 Functional connectivity analysis of experimental data

**Fig. 5:**
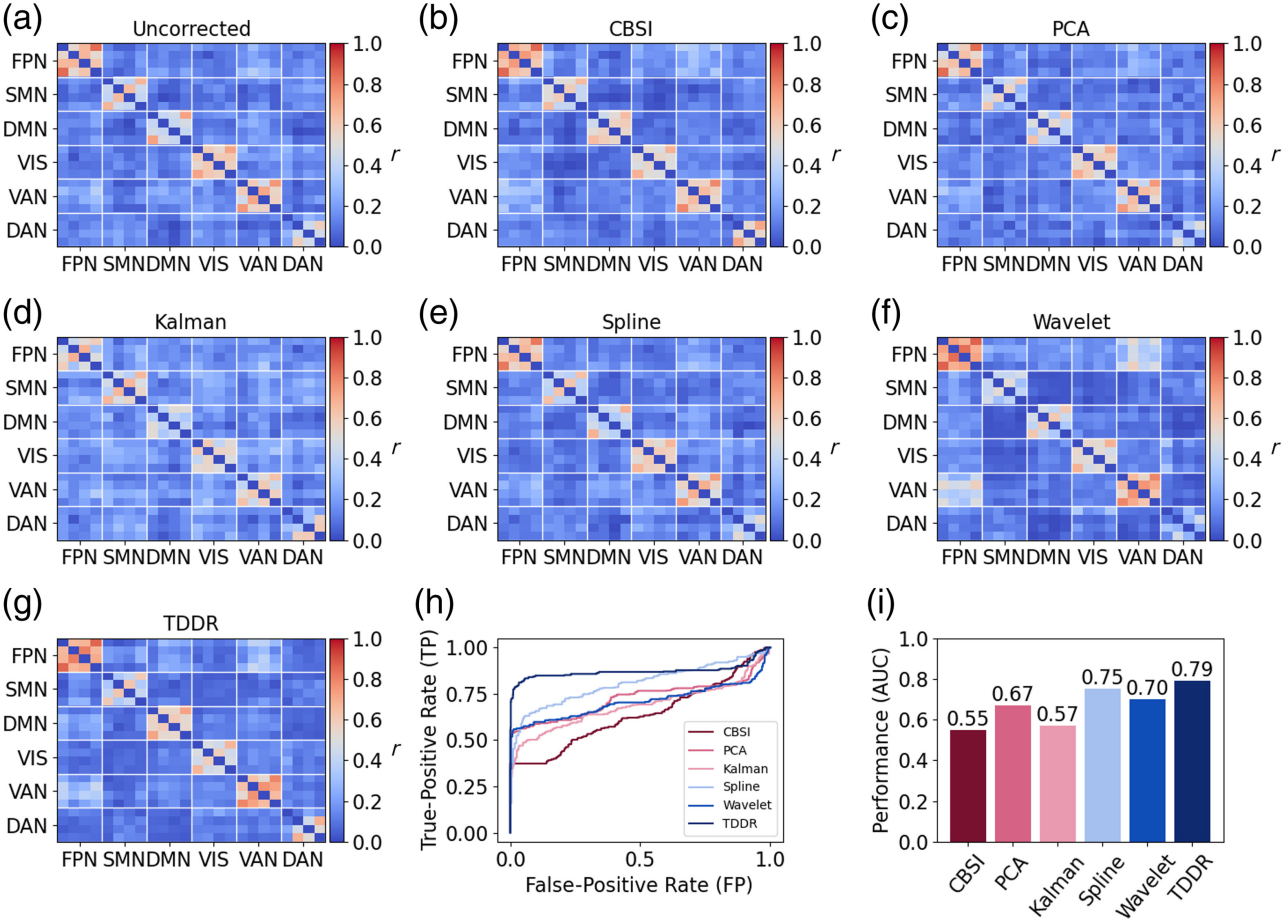
Visualization of the uncorrected and corrected functional connectivity (r) of each network in the experimental data (a)–(g). (h) and (i) Comparison of ROC and AUC obtained from different methods.

Similar to the simulation dataset, we observed strong within-network FC and weak between-network FC among all six networks [Figs. 5(a)–5(g)]. After MA correction, the within-network FC was effectively recovered for each network. The ROC analysis showed that TDDR achieved a mean AUC value of 0.79 [Fig. 5(h)], outperforming other methods such as spline interpolation (0.75), PCA (0.67), wavelet filtering (0.7), Kalman filtering (0.57), and CBSI (0.55) [Fig. 5(i)].

**Fig. 6:**
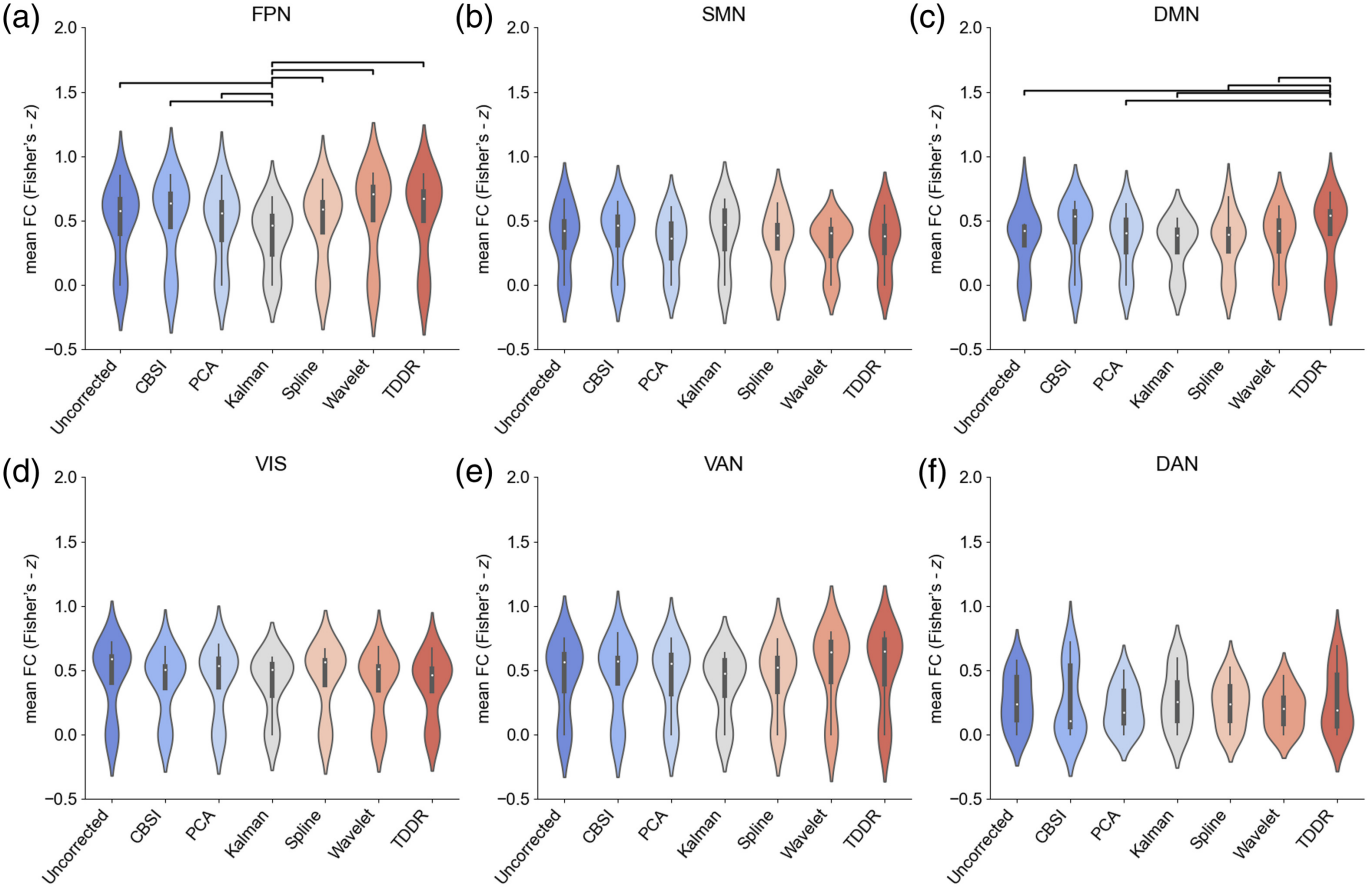
(a)–(f) Statistical comparison of the mean functional connectivity of each network before and after applying different MA correction methods in the experimental data. Each horizontal line indicates a significant difference between the two methods (p<0.05, FDR-corrected).

We also tested the mean FC of each network to evaluate the overall impact of various MA methods on these resting-state FC networks. As shown in Fig. 6, the mean FC z-value of the FPN network using Kalman was significantly lower than that of other groups (p<0.01). The mean FC z-value of the DMN using TDDR was significantly higher than other groups (p<0.01).

#### 3.2.2 Topological analysis

**Fig. 7:**
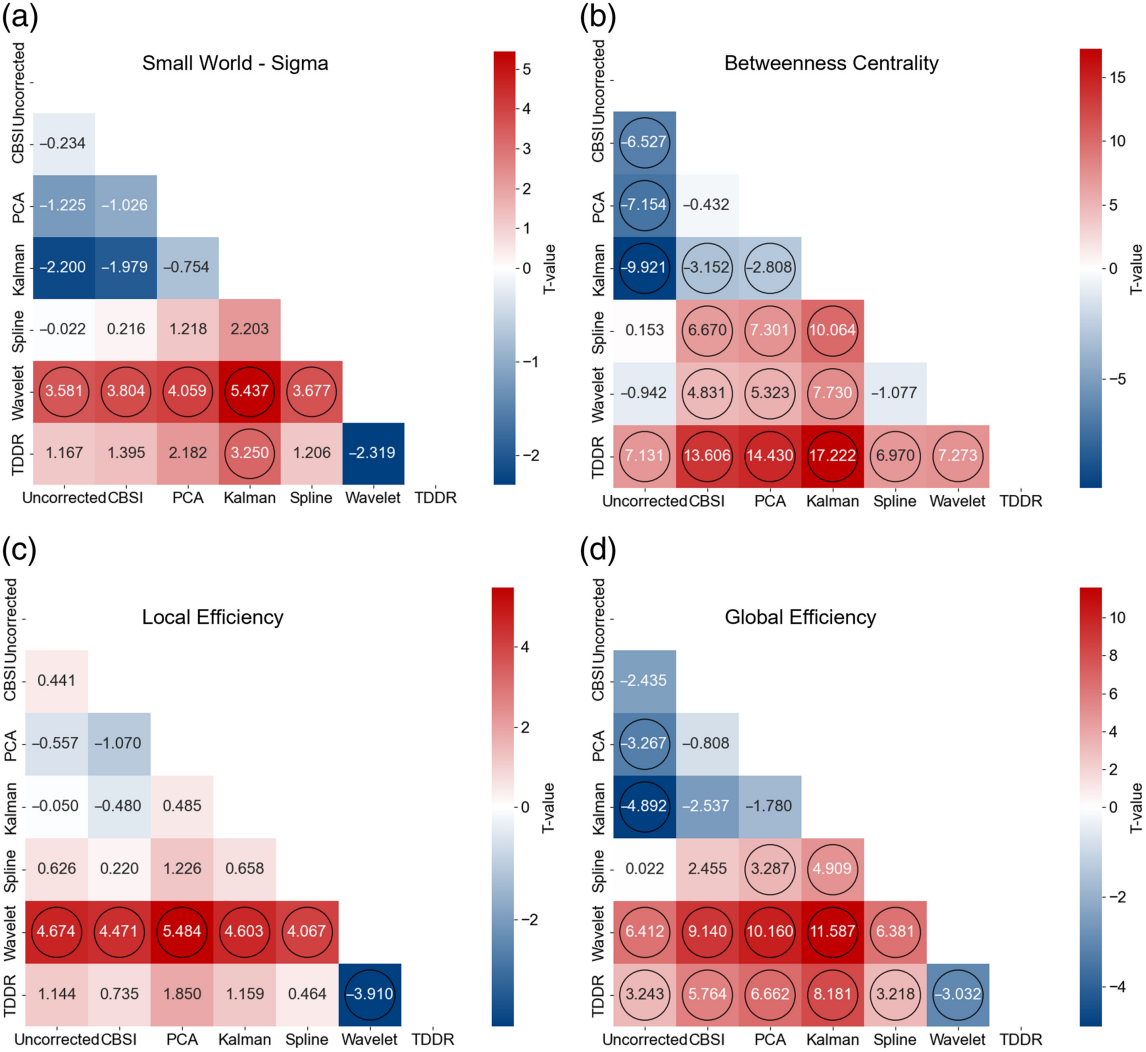
Statistical comparison of graph theory–based topological measures in the experimental data (y-axis versus x-axis). (a) Small world sigma. (b) Betweenness centralities. (c) Local efficiency. (d) Global efficiency. Each black circle indicates a significant difference between the two methods (p<0.05, FDR-corrected).

The impact of each MA correction algorithm on the graph theory–based metrics was examined through pairwise comparisons. For the small world, we found that wavelet filtering significantly enhanced this metric compared with other MA correction algorithms except the TDDR and uncorrected data [all p values<0.01, Fig. 7(a)]. In addition, TDDR significantly enhanced the measure of betweenness centrality compared with all other algorithms [all p<0.001, Fig. 7(b)]. Conversely, the Kalman filter appeared to significantly decrease the network betweenness centrality compared with all other algorithms [all p<0.001, Fig. 7(b)].

For the local and global efficiency, wavelet filtering significantly enhanced both metrics over other correction algorithms and uncorrected data [all p<0.001, Figs. 7(c) and 7(d)]. TDDR also significantly enhanced global efficiency compared with other algorithms except for the wavelet filtering [Fig. 7(d)]. Overall, both wavelet filtering and TDDR demonstrated consistently strengthened impact on all topological measures compared with other MA algorithms.

### 3.3 Exploratory Analysis of Motion Artifact Correction

The ratio of the number of remaining MAs after applying each method to the overall count of MAs before correction was calculated for comparative analysis of MA correction among the selected methods (Table S1 in the Supplementary Material). Overall, TDDR and wavelet filtering demonstrated superior performance in both the simulation and experimental datasets, with a MA removal rate exceeding 94%. In addition, representative HbO and HbR signals and their correlation coefficients obtained from the simulated and experimental datasets before and after the MA correction were analyzed. Although all methods performed well in the simulated dataset (Fig. S1 in the Supplementary Material), CBSI and Kalman filtering appeared to distort the experimental data after MA correction, as evidenced by the aberrant correlation coefficients between HbO and HbR signals (Fig. S2 in the Supplementary Material).

## 4 Discussion and Conclusion

Signal distortions due to the movements of the participants or lack of solid anchoring of the probes on the scalp typically impact the quality of fNIRS signals. It remains largely unknown how commonly used MA correction algorithms alter the analysis of FC and brain topology. In this study, we evaluated the impact of various MA correction approaches on the fNIRS-based FC and graph theory analysis through simulated and real experimental datasets. A comprehensive set of comparisons on FC property validated the effects of different MA removal algorithms on the network’s FC strength as well as topological property. Specifically, our findings highlighted the advantages of the TDDR and wavelet filtering algorithms for FC analysis. The present study shed light on the various effects of MA correction algorithms on brain FC analysis, and they might be used to identify the best method for FC analysis in future research.

The CBSI method assumes that the concentrations of HbO and HbR are strictly negatively correlated and that MAs diminish the negative correlation among the changes in the concentrations. Therefore, CBSI aims to eliminate the non-correlation introduced by artifacts while preserving the correlation owing to normal neuronal activity.^23^ However, the CBSI method’s performance in processing FC seems to be less favorable in both simulated and empirical datasets. This may be attributed to the fact that MAs typically induce a high degree of correlation among the variations in the two types of hemoglobin concentration, leading to an over-correction during CBSI adjustments.^39^ This over-correction not only eliminates artifacts but also potentially removes some of the correlated neuronal signals, resulting in a diminished FC. In addition, the correlation between HbO and HbR concentrations in the blood of the scalp during rest is not strictly negatively correlated,^40^ which may lead to worse performance in removing MAs in the resting-state signal and thus affecting the subsequent FC analysis.

PCA leverages the distinct temporal characteristics of artifacts and hemodynamic responses to delineate artifacts from variance explained in the data, resulting in a clarified post-PCA dataset. However, within this context, PCA is not deemed the optimal choice for processing FC. The basic principle of PCA lies in its multispectral approach, which necessitates the presentation of artifacts across multiple channels to identify MAs as principal components.^41^ Nevertheless, in actual dataset, minor artifacts restricted to a single channel, or limited regional channels may not be easily identified as principal components. In other words, PCA is effective at identifying global MAs. Furthermore, PCA operates by directly eliminating the components with the greatest variance within the data, which may concurrently encompass hemodynamic fluctuations. Owing to potential reasons, PCA correction might attenuate the correlated components among multiple channels within the same network, thereby influencing the results pertaining to FC and graph theoretical properties. Although PCA is an effective tool for minimizing artifacts in fNIRS datasets, we suggest that it needs to be used with caution when conducting FC analysis, with full consideration of its underlying assumptions and its potential impact on the data.

Spline interpolation applies a methodology that first identifies data segments affected by artifacts and then constructs new data points through smooth functions interpolated among data points unaffected by artifacts, eventually replacing the impacted data segments with the smooth spline function to eliminate interference. Although spline interpolation has been demonstrated to effectively remove MAs,^17^ our study did not conclusively establish that spline interpolation is the foremost method for FC analysis. Spline interpolation has also been critiqued in the context of traditional motion correction for time trace data particularly when considering the nuances and specific demands.^11^^,^^20^^,^^42^ In real datasets, the mean FC and AUC indicate that spline interpolation might not be the optimal choice. There are multiple factors contributing to these findings. In previous literature, the efficacy of spline-based artifact removal heavily relied on the accuracy of artifact detection prior to correction; if artifact detection is imprecise, the outcomes could be unstable.^4^ Furthermore, subtracting a smooth spline might also inadvertently remove some genuinely relevant hemodynamic responses.^43^ A crucial consideration is that although certain artifacts may be erroneously interpreted as FC among brain regions, eliminating misleading signals can reduce the incidence of false positives. However, incomplete or incorrect removal of artifacts may result in erroneous interpretations of FC among brain areas. These factors could potentially explain why spline interpolation analysis within networks for FC is not the most advocated approach in this paper.^44^

Our findings indicate that the Kalman filtering method may be the least effective artifact correction technique in this context. The primary application of the Kalman approach is orchestrated in two stages: during the prediction phase, it leverages a known system model to anticipate the subsequent state and associated uncertainties, and in the update phase, new measurement data are assimilated into the prediction, thereby refining the state estimation.^45^ This method not only offers an optimized estimation of the state but also enhances the stability of artifact removal.^46^ However, the results produced by the Kalman method in both simulated and experimental data processing substantially diverge from those obtained through alternative techniques.^14^ Consequently, in contrast to the results observed with other methodologies, it fails to recover the intrinsic strong FC within networks. Several potential reasons might account for the inability of FC processes to capitalize on the Kalman filter. Firstly, the presence of numerous non-Gaussian noise components within MAs could lead to a misalignment between the model and actual data, detrimentally affecting the accuracy of the model. Moreover, the nonlinear characteristics associated with Kalman processing might precipitate a decline in the performance of the filter, thereby compromising the precision of FC. Optimizing and validating parameters are crucial for maintaining the accuracy and reliability of FC. Consequently, addressing the challenge of preventing the incorrect exclusion or elimination of signal components associated with brain activity could be a significant research focus for the further refinement of the Kalman filtering.^47^

Both wavelet filtering and TDDR performed relatively well in the simulated and experimental data. Specifically, the wavelet filtering removes the spikes in the signal effectively, but it also aggravates the baseline shift of the signal, especially in the parts of the signal containing serial disturbance.^48^ In contrast, PCA leaves residuals when dealing with such perturbations, and the Kalman method causes baseline shifts owing to the residual artifacts. In addition, the TDDR method corrected most of the artifacts contaminated in the signal. Through robust regression with iterative reweighting techniques, the TDDR approach has been demonstrated to effectively remove MAs without the requisition of user-defined parameters. In our study, the effectiveness of this approach for FC analysis was confirmed by the simulation experiment that demonstrated the superior performance of TDDR in detecting activation states that are almost identical to those of motion-free data, which is consistent with the existing literature for brain mapping analysis.^42^ The evaluations on the experimental dataset further demonstrated that TDDR could produce stronger and broader activation effects than any other method. Furthermore, wavelet filtering significantly increased the performance of graph theory–based metrics compared with other methods, especially enhancing the depiction of small-world properties, local efficiency, and global efficiency. We can infer that wavelet filtering performs well in both FC analysis and topological analysis and may help to accurately assess the global network structure of the brain and its functional efficiency. Taken together, the TDDR and wavelet filtering methods offer an advantageous ability in MA removal, facilitating the restoration of network functional correlations that may be submerged by MAs. Meanwhile, these algorithms are designed to preserve the integrity of the signal, ensuring that the MA correction does not lead to distortion or confusion in the brain network property.

Several limitations should be acknowledged in our study. First, all artifacts in the simulation study were randomly generated and added to the fNIRS signals according to previous studies.^13^^,^^19^^,^^49^^,^^50^ Although we meticulously considered the mathematical properties of real-world data, it is essential to acknowledge that simulations might not fully capture the complexity of actual scenarios. Real-world measurements often involve more intricate artifacts, such as simultaneous spikes, shifts, and other irregularities, which were not explicitly modeled in our simulation. Consequently, evaluating the effectiveness of various MA correction methods under these multifaceted conditions warrants further extensive investigation. In addition, we only computed FC using Pearson’s correlation coefficient. This traditional metric primarily captures linear relationships among brain regions, which may not adequately account for the intricate and possibly non-linear interactions among brain regions. In addition, our analysis assumed that FC within predefined neural networks is consistently robust, whereas connectivity among separate networks is inherently weaker. This hypothesis draws inspiration from patterns observed in other neuroimaging modalities (e.g., fMRI). Nevertheless, the stratification between strong intra-network and weak inter-network FC in the context of fNIRS has not been sufficiently substantiated through empirical evidence. As such, there is an implication for fNIRS-based studies in this domain to engage in a more critical examination of these assumptions and to rigorously validate the FC patterns elicited within and between neural circuits in the future. Another limitation of this study is that only a single MA correction method was assessed. Recent studies showed that a combination of two or more MA correction methods could achieve better performance in fNIRS data analysis.^39^^,^^51^^,^^52^ Two-step approaches, such as combining TDDR and wavelet filtering techniques can be explored in future FC studies. In particular, TDDR can be used to capture the dynamic changes of artifacts in the time domain, whereas wavelet filtering can provide high-resolution noise removal in the frequency domain, achieving more reliable FC analysis. Hybrid techniques such as weighted methods can also be considered, in which various algorithms are integrated using weighted averaging or combining regression models with MARA.^53^ This will allow for a comprehensive correction that takes into account various sources of artifacts. In addition, deep learning-based methods, such as the deep adaptive ensemble architecture, were recently proposed for motion artifact removal in fNIRS signals.^13^ Such methods may yield superior ability over conventional methods by significantly reducing residual motion artifacts, lowering mean squared error, and enhancing computational efficiency. However, the present study did not include these methods for evaluation because deep learning methods in fNIRS research are still relatively rare and can be challenging to implement due to significantly higher dependency on computational resources and complicated parameter tuning.

## Supplementary Material



## Data Availability

The data and scripts that support the findings of this article are available from the authors upon request.
