# Supplementary material for: Disentangling the impact of motion artifact correction algorithms on functional near-infrared spectroscopy–based brain network analysis
Source: Neurophotonics. 2024 Oct 23;11(4):045006. doi: 10.1117/1.NPh.11.4.045006 (PMC11498316; doi:10.1117/1.NPh.11.4.045006)
Supplement: Supplementary file 1 [file NPh_011_045006_SD001.pdf]

# Disentangling the impact of motion artifact correction algorithms on fNIRS-based brain network analysis

**Table S1. The performance of motion artifact (MA, in %) removal of each MA removal algorithm. We employed the *hmrMotionArtifact* from the HOMER2 NIRS processing package to detect motion artifacts before and after the processing. *Percentage % = (the number of MAs after MA removal / the total number of MAs before MA removal) *100%.***

| | CBSI | PCA | Kalman | Spline | Wavelet | TDDR |
|---|---|---|---|---|---|---|
| Simulated data | 2.03% | 8.75% | 9.84% | 6.72% | 4.45% | 1.97% |
| Experimental data | 16.67% | 21.67% | 35.00% | 11.67% | 3.33% | 5.50% |

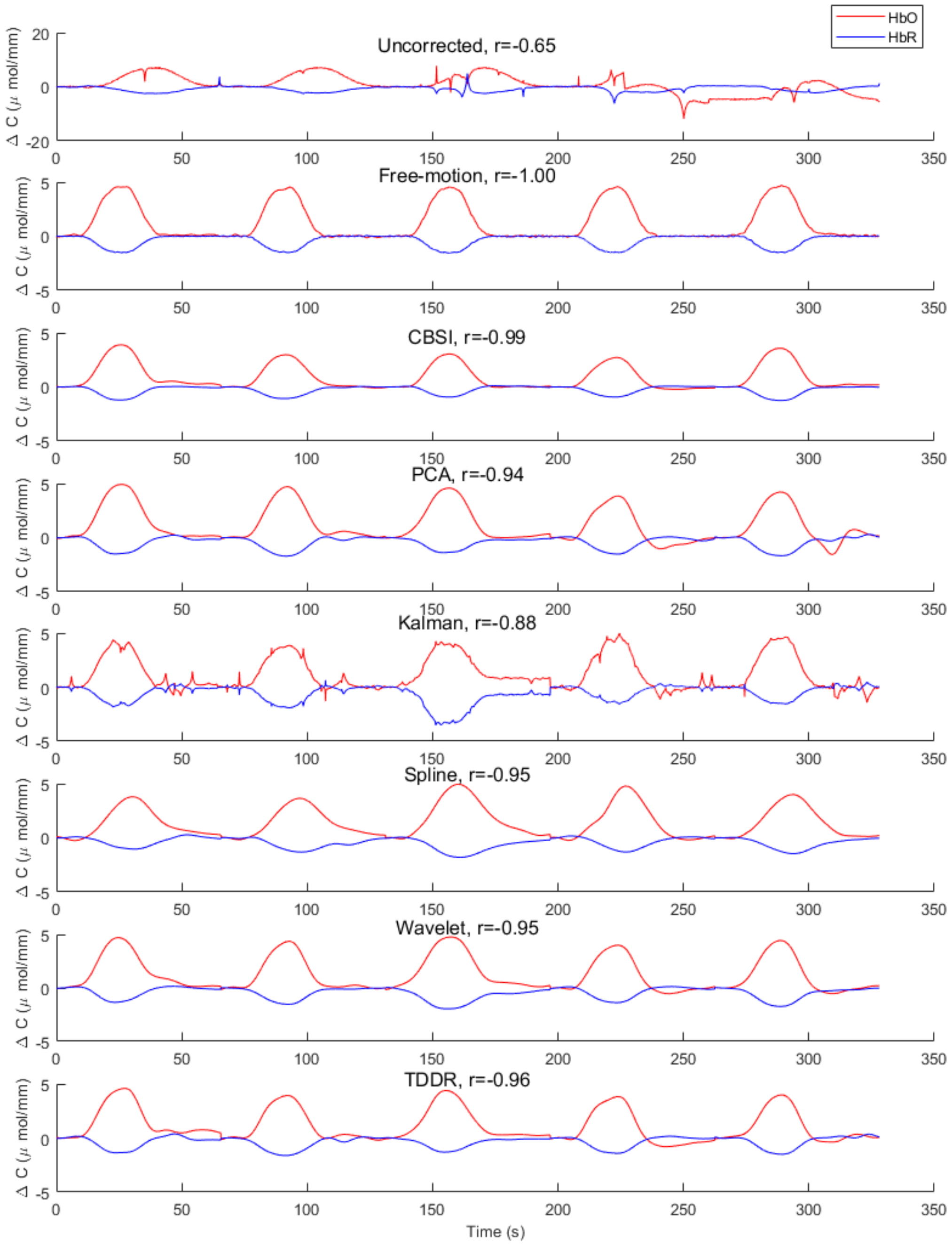


**Fig.S1. Demonstration of representative HbO and HbR signals before and after MA correction on the simulation data. The change in concentration of HbO (red) and HbR (blue) and the correlation (r) are shown for an uncorrected signal, motion-free signal, and the signals after MA correction using CBSI, PCA, Kalman, spline interpolation, Wavelet filtering, and TDDR.**

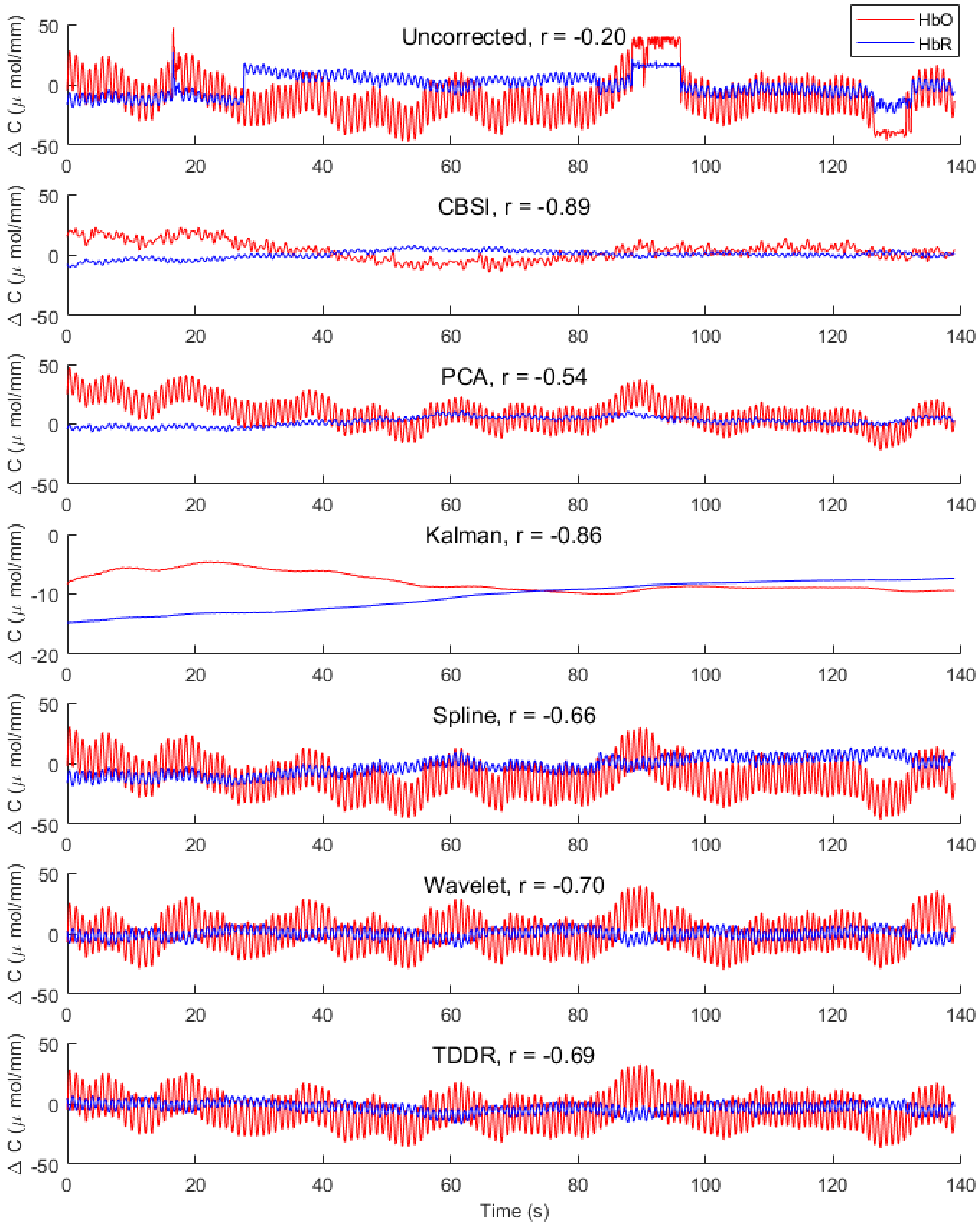


**Fig.S2. Demonstration of representative HbO and HbR signals before and after MA correction on the experimental data. The change in concentration of HbO (red) and HbR (blue) and the correlation (r) are shown for an uncorrected signal, and the signals after MA correction using CBSI, PCA, Kalman, spline interpolation, Wavelet filtering, and TDDR.**
